# Older adults ‘s hospitalizational costs and burden study in China——analysis from CHARLS data 2018

**DOI:** 10.3389/fpubh.2024.1418179

**Published:** 2024-07-25

**Authors:** Shanheng He, Ying Bian

**Affiliations:** ^1^Institute of Chinese Medical Sciences, University of Macau, Macau, China; ^2^State Key Laboratory of Quality Research in Chinese Medicine, University of Macau, Macau, China; ^3^Department of Public Health and Medicinal Administration, Faculty of Health Sciences, University of Macau, Macau, China

**Keywords:** China, older adult people, medical expenditures, hospitalizational costs, hospitalizational burden

## Abstract

**Objective:**

The aging Chinese population is driving up health care costs, with hospitalizational accounting for a large portion of total health care costs. By 2012, hospitalization costs for people over 60 years of age exceeded outpatient costs, marking a change in the allocation of medical resources. Further research is needed on the factors influencing changes in hospitalizational costs and burden. This paper examines the costs and burden of hospitalization for older adults from a micro perspective, providing new evidence to explain how social, medical, family, personal, and geographic factors affect them.

**Methods:**

Utilizing data from the 2018 China Health and Retirement Longitudinal Study (CHARLS), a linear regression model was constructed to investigate the impact of various factors on the hospitalization costs and burden among the older adult in China. To ensure the heterogeneity of the results, the sample was divided into subgroups based on different regions for comparative analysis. Additionally, collinearity among the variables was examined.

**Results:**

The average hospitalization costs for the older adult are $1,199.24, with a burden score of 0.5. Residence, type of chronic diseases, region, family size, type of health service facility, received distance, smoke and alcoholic significantly affect the out-of-pocket expenses for older adult hospitalizations. In terms of the burden of hospitalization for the older adult, Residence, health insurance, education, type of chronic diseases, region, family size, ethnic, type of health service facility, received distance, smoke, alcoholic and pension significantly impact the hospitalization burden for the older adult.

**Conclusion:**

This paper provides a new perspective to explain the factors influencing hospitalizational costs and burden in China. The policy recommendations include expanding health insurance coverage and promoting commercial insurance to enhance the accessibility and financial security of healthcare services. Strengthening primary care is suggested to reduce the burden on hospitals and lower the overall cost of hospitalization. Policies aimed at addressing regional healthcare disparities are proposed, along with targeted support for vulnerable groups, including subsidies and culturally sensitive services.

## 1 Introduction

The improvement of living standards and better medical conditions have led to a decline in the birth rate and an extension of life expectancy, gradually transitioning China into an aging society. Since the 1980s, the proportion of the population aged 0–14 has been decreasing, while the proportion of those aged 65 and above has been steadily increasing. In the year 2000, individuals aged 60 and above constituted 10% of the total population in China, marking the country’s entry into the internationally recognized stage of an aging society. According to the sixth national population census conducted in 2010, the proportion of people aged 60 and above exceeded 13.26% of the total population. By the time of the seventh ethnical population census in 2020, the percentage of the population aged 60 and above had risen to 18.7% of the total population. This is a notable increase from the 7% recorded in 2000. It indicates that within a span of 21 years, the proportion of China’s older adult population aged 65 and older doubled from 7 to 14%. In comparison, it has typically taken high-income countries (HICs) around 55 years to achieve a similar doubling of this demographic segment (1–4). It is projected that by the year 2050, the proportion of the population aged 65 and above will reach 26%, with the proportion of those aged 80 and above accounting for 8% (5). These findings indicate that China has long entered the aging period, and the degree of aging is becoming increasingly severe.

Since the beginning of the 21st century, healthcare expenditure in the People’s Republic of China has been on the rise. The *per capita* healthcare expenditure soared rapidly from 1,407.74 Yuan (CNY) in 2008 to 3,783.83 Yuan (CNY) in 2017 (6). Concurrently, the proportion of government health expenditures increased from 5.7 to 7.5%, and as a percentage of the overall gross domestic product (GDP), it rose from 1.1 to 1.8% (7). Aging is a pivotal determinant influencing healthcare expenditure. While the aggregate level of public health spending is largely driven by societal determinants, its distribution is significantly dictated by health and disability. Consequently, additional increments in medical innovation and healthcare spending are often targeted towards the older adults, exacerbating the impact of population aging (8, 9). Evidence from Beijing indicates that the age effect on healthcare expenditure is most pronounced among the population aged 65 or over. The *per capita* healthcare expenditure for individuals aged 65 years or older is 7.25 times that of the population aged under 25 years, 1.61 times that of the population aged 25 to 59 years, and 3.47 times that of the population aged 60 to 64 years (10).

Hospitalization costs represent a substantial portion of overall medical expenses. According to the OECD Health Statistics 2021 report, inpatient care accounts for 28% of healthcare expenditure in OECD countries. From 2009 to 2013, the annual growth rate of hospitalization expenditure was 0.3%, while from 2013 to 2019, it increased by 2.1%. The expenditure and burden of hospitalization reflect the condition of hospitalized patients (11). The higher the hospitalization costs and burden, the higher the economic risk of illness for patients. Understanding the proportion of hospitalization costs in healthcare spending and its growth trend is essential for assessing the overall costs of the healthcare system and can serve as a basis for allocating medical resources.

In China, existing research has shown that the total outpatient expenses for individuals aged 60 and above were 1056.44 billion yuan in 2009, while the total hospitalizational costs were 957.02 billion yuan. By 2012, hospitalizational costs had increased to 2132.10 billion yuan, surpassing outpatient expenses, which totaled 2073.88 billion yuan (12). This reversal in expenditure highlights the shifting dynamics and patterns in healthcare utilization and resource allocation, underscoring the need for further investigation into the factors driving changes in hospitalization costs and burden.

The research by De Foor (13) indicates that there is a significant difference in hospitalization costs between older adult men and women, with men incurring higher hospitalization costs than women. Research by Zhang Rong and Li Fang demonstrates that medical insurance significantly increases outpatient and inpatient medical expenses for older adult individuals with chronic diseases. China’s health insurance system has been effective in increasing health care utilization and reducing out-of-pocket (OOP) hospitalization expenditures (14). China’s medical insurance system has successfully facilitated greater access to healthcare services and decreased out-of-pocket expenses for hospital stays (15, 16). According to the research by Ye Ziyi et al. variations exist in hospitalization costs among older adult patients of different genders, ethnicities (2–4). Ouyang Jing and colleagues have also pointed out that there are statistically significant differences in hospitalization costs among older adult patients across different genders, ethnicities, and healthcare institutions visited (17). The level of medical facilities also affects inpatient medical expenses, and the rational utilization and distribution of town-level and county-level hospitals may help to reduce medical costs (18). The study conducted by Yanghe et al. (19) indicates that there are statistically significant differences in the average hospitalizational costs and burden among patients with different educational levels, marital statuses, and types of medical insurance. The size of the household, origin from different regions, as well as the distance to medical facilities, have been shown to influence expenses (20, 21). Additionally, for chronic diseases and their types, many scholars have included them in the scope of factors affecting hospitalization costs (21, 22). Lifestyle and income are also significant factors influencing older adult hospitalization costs (23).

## 2 Research program

### 2.1 Data sources

This study used data from The China Health and Retirement Longitudinal Study (CHARLS) database. CHARLS is an ethnically representative population-based survey designed to study social, health, and economic issues of residents aged ≥45 years in light of the burgeoning aging population in China (24). Using the Probabilities Proportional to Size (PPS) sampling method, by adopting regional status, urban-rurality, and gross domestic product data in 2009 as stratified indicators, CHARLS followed the top-down county-neighborhood-household-individual order procedure in sampling. For sample weighting, CHARLS constructed sample weights for households, individuals, and biomarker data directly from the sampling probabilities (24). Face-to-face computer-assisted personal interviews (CAPI), physical measurements, and blood tests were conducted every 2 years to collect data. This study used data from 2018 and covered 17,970 residents in 10,524 households in 28 provinces in China, with a response rate of 83.84%.

According to the current legal retirement age in China, we have taken the definition of the older adult as 60 years old for men and 55 years old for women.

### 2.2 Research hypothesis

Rongfei et al. (25) research shows that in 2010, the provinces with the highest inpatient medical expenses in China were also twice as high as the lowest. Empirical studies have illustrated that in 2017, the maximum *per capita* hospitalization expenditure was 3.64-fold that of the minimum, a disparity that increased to 3.79-fold by 2019. This indicates a secular upward trend with significant variability in *per capita* costs across different regions. Spatial analysis of *per capita* hospitalization costs has revealed a pattern of geographic concentration, with a pronounced disparity characterized by higher costs in the eastern coastal regions compared to the western regions of China (26).

In terms of impact on the medical burden of the older adult, Chen et al. (23) made a systematic regression review to identify the factors that affect the medical expense burden of the older adult population and studied these factors from different angles. Various factors have been identified as having a significant impact on changes in health costs for older adults, including social, medical, family, and personal factors (23).

Accordingly, the design idea of the study is to first analyze the hospitalizational costs and burden of the older adults in various provinces, and then select the social, medical, family and personal factors that affect the hospitalization costs and burden of the older adult in the data sources. Due to the impact of residency and region on costs and burdens, the fifth factor, geographic factors, is selected. OLS regressions are used to determine how they affect costs and burdens. In the study, we took the exchange rate value of December 2018 and discounted it to 2023, where 1 US dollar is equal to 6.51 yuan.

### 2.3 Study variables

In our study, we focused on older adults’ hospitalizational costs and burden in 2018 as the outcome variable. The hospitalizational costs of the sample are measured by asking ‘What is the total cost of hospitalization in the past year (excluding escort, family transportation and accommodation)?’ ‘How much of it was self-paid?’. The hospitalizational costs are the out-of-pocket (OOP). The hospitalizational burden is constituted by the ratio of self-payment expenses to total expenses. Because personal hospitalization costs generally have a positively skewed distribution. So, take the natural logarithm of it. If out-of-pocket medical costs are 0, still take 0.

The explanatory variables encompass social factors such as health insurance and pension, medical factors including the type of health service facility and received distance, family factors like family size, and personal factors which involve gender, education, chronic disease, type of chronic diseases, marital status, ethnicity, smoking status, and alcohol consumption. Additionally, geographic factors are considered, specifically focusing on residence and region. The variable definitions and descriptions are as shown in Table 1.

**Table 1:** Definition of variables.

Variable	Variable definition and assignment	
Hospitalizational costs	Measured by asking ‘What was the out-of-pocket medical cost for all the inpatient care you received during the past year?’	
Hospitalizational burden	The ratio of out-of-pocket medical costs to total costs.	
Residence	0 for Rural; 1 for Urban	
Gender	0 for Male; 1 for Female	
Health insurance	Basic health insurance	No health insurance is the baseline
	Other health insurance	
Education	“0” is Under primary school education; “1” refers to those who have received elementary school; “2” is the Middle school education; “3” is a Above middle school.	
Chronic disease	0 for no; 1 for yes	
Type of chronic diseases	Hypertension	0 for no; 1 for yes
	Dyslipidemia	
	Diabetes	
	Malignancy	
	Chronic lung disease	
	Liver disease	
	Heart attack	
	Stroke	
	Kidney disease	
	Stomach or digestive disorders	
	Emotional and mental problems	
	Memory-related disorders	
	Arthritis or rheumatism	
	Asthma	
Region	Central areas	Eastern areas are the baseline
	Western areas	
	Northeast areas	
Family size	The people who live with the interviewee and the interviewee jointly form a Household member and share the household income and expenses.	
Marital status	Unmarried, divorced (separated as a spouse, divorced, widowed) is “0,” married is “1.”	
Ethnic	0 for Other; 1 for Han	
Type of health service facility	General hospital	Chinese Medicine Hospital is the baseline
	Specialized hospital	
	Township Hospital	
	Other	
Received distance	The distance from the interviewer’s home to the last outpatient medical facility	
Smoke	0 for Never smoke; 1 for Now or have smoked	
Alcoholic	0 for Haven’t drunk alcohol in the past year; 1 for Have drunk alcohol in the past year	
Pension	0 for interviewees who participated in or receive benefits; 1 for interviewees who not participated in or receive benefits	

## 3 Empirical analysis

### 3.1 Descriptive statistics

The Table 2 presents a comprehensive analysis of hospitalization costs incurred by older adult individuals in China during the year 2018. It provides detailed statistics categorized by various demographic and socioeconomic variables, shedding light on the intricate patterns of healthcare expenditure among this population segment.

**Table 2:** Hospitalizational costs and burden of the older adults of China in 2018.

Variables		Number	Total costs		OOP of inpatient		Burden	
			Mean	SD	Mean	SD	Mean	SD
All		2,254	2518.83	4980.29	1199.24	2428.75	0.50	0.28
Gender	Male	1,016	2732.60	5989.91	1201.82	2522.22	0.48	0.28
	Female	1,238	2343.39	3958.17	1197.13	2350.30	0.51	0.28
Residence	Rural	1,270	2122.60	3994.98	1141.11	2450.91	0.53	0.29
	Urban	984	3030.21	5981.67	1274.26	2399.01	0.46	0.27
Health insurance	Basic health insurance	2,160	2523.81	5028.40	1205.34	2449.78	0.50	0.28
	Other health insurance	56	2633.30	3118.87	948.35	1307.35	0.41	0.31
	No health insurance	38	2067.08	4497.47	1222.50	2522.41	0.79	0.35
Education	Illiterate	715	2214.98	3880.58	1102.89	2195.13	0.53	0.29
	Did not Finish Primary School	466	2274.40	4755.27	1163.38	2704.76	0.53	0.27
	Elementary School	536	2649.88	4429.20	1300.26	2309.33	0.50	0.27
	Middle School	319	2904.80	7019.35	1179.41	2544.56	0.43	0.26
	Above middle School	218	3150.87	6152.27	1372.53	2643.37	0.44	0.29
Chronic disease	Yes	2,158	2555.34	5049.16	1213.24	2448.36	0.50	0.28
	No	96	1698.11	2944.65	884.45	1919.51	0.50	0.29
Type of chronic diseases	Hypertension	1,275	2634.67	5282.26	1242.89	2487.22	0.50	0.28
	Dyslipidemia	774	2759.27	5971.68	1257.03	2642.68	0.49	0.28
	Diabetes	498	2992.28	5170.46	1336.91	2505.55	0.47	0.27
	Malignancy	119	7625.51	13480.41	3581.88	5270.70	0.53	0.29
	Chronic lung disease	650	2707.67	4709.12	1240.30	2289.36	0.49	0.29
	Liver disease	239	3419.36	8766.98	1486.33	2989.25	0.53	0.29
	Heart attack	883	2685.92	5838.91	1219.85	2457.80	0.48	0.28
	Stroke	418	2747.68	4179.31	1314.29	2458.05	0.48	0.28
	Kidney disease	398	3319.55	7780.01	1423.92	2796.01	0.47	0.27
	Stomach or digestive disorders	953	2393.87	4285.55	1178.67	2345.32	0.51	0.28
	Emotional and mental problems	107	2801.08	5737.31	1481.02	2984.47	0.47	0.28
	Memory-related disorders	230	2801.47	5343.23	1208.62	2257.19	0.47	0.29
	Arthritis or rheumatism	1,184	2287.07	4110.11	1122.90	2120.73	0.51	0.29
	Asthma	309	2574.47	4907.72	1090.42	2004.03	0.47	0.28
Region	Eastern areas	452	3138.66	5263.93	1603.45	2869.47	0.53	0.28
	Central areas	688	2236.61	4380.82	952.13	1783.25	0.48	0.28
	Western areas	972	2359.10	4296.66	1156.36	2478.90	0.50	0.29
	Northeast areas	142	3006.60	9220.52	1403.38	3038.38	0.49	0.26

In the 2018 cohort of hospitalized patients in China, females constituted a larger proportion at 55.0% (1,238/2254), while males accounted for 45.0% (1,016/2254). Regarding residential areas, rural patients made up 56.5% (1,270/2254) of the sample, with urban patients comprising 43.5% (984/2254). The vast majority of patients were covered by basic health insurance, with 95.8% (2,160/2254) having access, while a small fraction had other types of health insurance (2.5%, 56/2254) or no health insurance at all (0.7%, 38/2254). In terms of educational attainment, 31.7% (715/2254) of the patients were illiterate, 20.7% (466/2254) did not complete primary school, 24.2% (536/2254) had completed primary school, 14.2% (319/2254) had completed secondary school, and only 9.7% (218/2254) had attained education levels beyond secondary school.

Among the hospitalized patients, 95.7% (2,158/2254) suffer from at least one chronic disease. Hypertension was the most prevalent, affecting 56.4% (1,275/2254) of the patients, followed by arthritis or rheumatism (52.5%, 1184/2254) and gastrointestinal diseases (42.3%, 953/2254). Other common chronic conditions include heart attack (39.2%, 883/2254), dyslipidemia (34.4%, 774/2254), chronic lung disease (28.9%, 650/2254), diabetes (22.1%, 498/2254), stroke (18.5%, 418/2254), kidney disease (17.7%, 398/2254), asthma (13.7%, 309/2254), liver disease (10.6%, 239/2254), memory-related disorders (10.2%, 230/2254), malignancy (5.28%, 119/2254), and emotional and mental problems (4.7%, 107/2254).

The regional distribution of hospitalized patients revealed that the western region had the highest proportion of patients, at 43.3% (972/2254), followed by the central region (30.5%, 688/2254), the eastern region (20.1%, 452/2254), and the northeastern region (6.3%, 142/2254). These data reflect the health status and medical needs of the older adult population across different regions in China, providing crucial insights for the development of public health policies and the allocation of resources.

The average total cost for hospitalization among the older adult is $2518.83, with a notable standard deviation of $4980.29, indicating a wide range of expenses within this demographic group. On average, out-of-pocket (OOP) expenses amount to $1199.24, with a standard deviation of $2428.75, suggesting significant variability in the financial burden borne by older adult patients. Male older adult individuals tend to incur slightly higher average total costs ($2732.60) and OOP expenses ($1201.82) compared to their female counterparts, although the difference in burden is marginal. Urban older adult individuals generally face higher average total costs ($3030.21) and OOP expenses ($1274.26) compared to their rural counterparts. However, rural elders exhibit a slightly higher burden. Older adult individuals covered by basic health insurance schemes report higher average total costs ($2523.81) and OOP expenses ($1205.34) compared to those with other forms of health insurance or without coverage. However, the burden is relatively lower for the former group. The level of educational attainment among older adult individuals correlates with healthcare expenses, with those with lower educational levels (e.g., illiterate, or incomplete primary education) experiencing lower average total costs and OOP expenses but bearing a higher burden. Older adult individuals diagnosed with chronic diseases generally face higher average total costs and OOP expenses compared to those without such conditions. However, the burden remains relatively consistent across both groups. Different chronic diseases exhibit varying impacts on healthcare expenses. For instance, malignancy is associated with significantly higher expenses compared to other chronic conditions. Older adult individuals residing in eastern regions incur substantially higher average total costs and OOP expenses compared to their counterparts in other regions, leading to a relatively higher burden.

### 3.2 Older adult’s hospitalizational costs and burden in each province

In China, the relationship between the ranking of *per capita* GDP and the out-of-pocket expenses and burden for older adult residents’ hospitalizations across various provinces provides a critical perspective for understanding the link between economic development and the affordability of healthcare services. Comparative analysis reveals some striking patterns.

**Figure 1:**
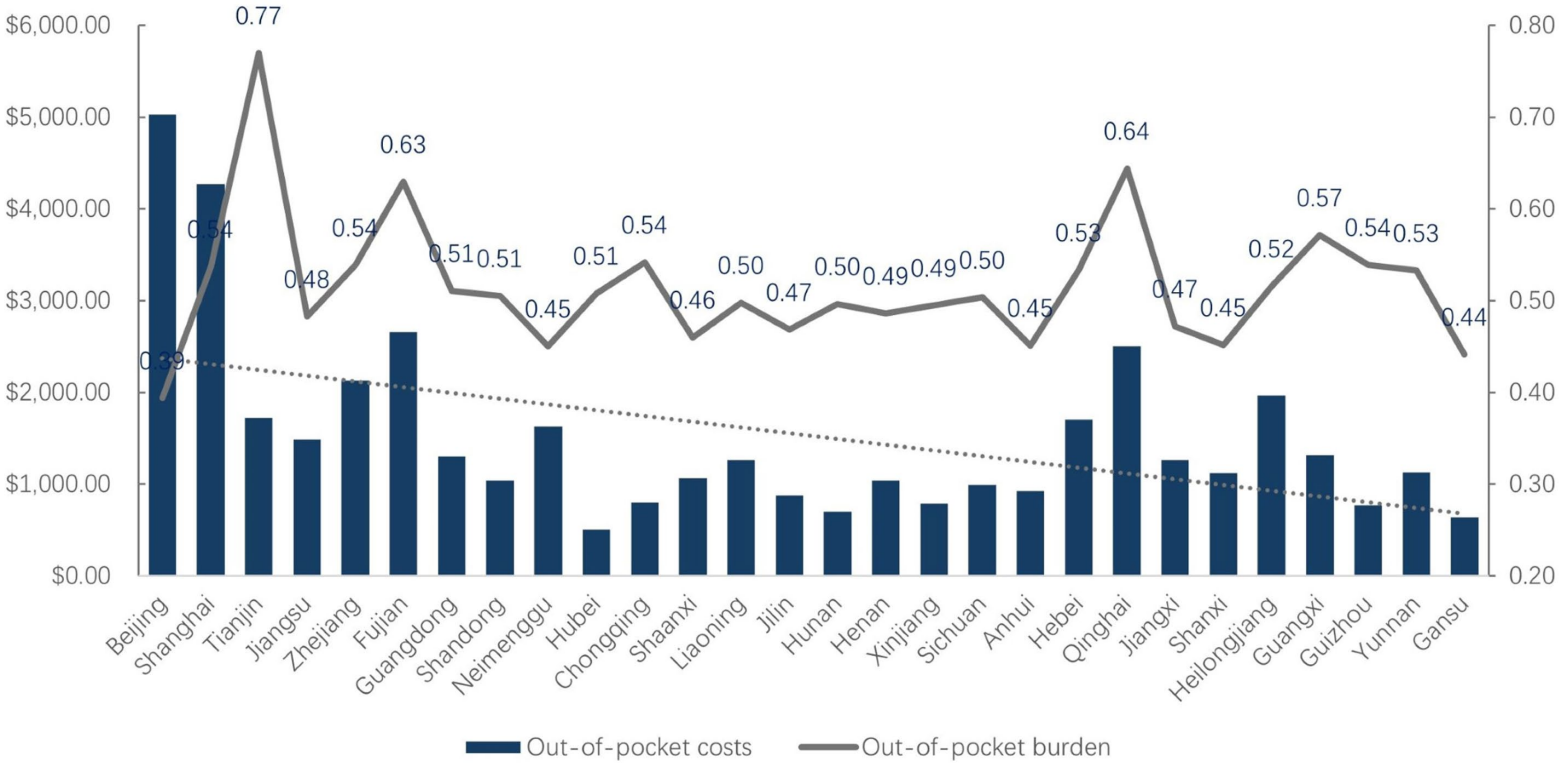
Older adult’s hospitalizational costs and burden in each province.

This is shown in Figure 1, the data indicates significant disparities in the out-of-pocket medical burden among different provinces. Beijing has the lowest proportion of medical burden, at 0.39, with out-of-pocket expenses for hospitalization amounting to $5027.92. Shanghai’s medical burden ratio is 0.54, with out-of-pocket hospitalization costs at $4273.09. In contrast, Tianjin has the highest medical burden ratio, reaching 0.77, yet the out-of-pocket expenses are relatively lower, at $1724.59. Furthermore, provinces such as Jiangsu, Zhejiang, and Guangdong, despite their high GDP rankings, do not exhibit the lowest ratios of medical burden. This may reflect the higher cost of medical services in these regions. On the other hand, provinces like Guizhou, Yunnan, and Gansu, which have lower GDP rankings, do not necessarily have the highest ratios of medical burden, possibly due to local living costs, healthcare policies, and residents’ health needs.

Overall, it can be observed from the data that as the ranking of *per capita* GDP decreases, the *per capita* out-of-pocket expenses for older adult hospitalization tend to decrease as well, while there is no distinct trend in the *per capita* hospitalization burden, which mostly hovers around 0.5.

This analysis underscores the complexity of the relationship between economic development and healthcare affordability. It suggests that while economic prosperity generally correlates with lower out-of-pocket medical expenses, other factors, including healthcare policies and the cost of living, also play significant roles in determining the medical burden on the older adult population. Policymakers may need to consider these multifaceted factors when designing interventions to improve healthcare affordability and accessibility for the aging population across different economic settings.

### 3.3 Multicollinearity test

The Table 3 presents the results of multicollinearity diagnostics through the calculation of Variance Inflation Factors (VIF) and their reciprocals (1/VIF). These metrics assess the degree of collinearity among the independent variables in a regression model. In regression analysis, multicollinearity occurs when independent variables are highly correlated with each other, which can lead to unreliable coefficient estimates and inflated standard errors. VIF measures the extent to which the variance of an estimated regression coefficient is increased due to collinearity (27).

**Table 3:** Results of multicollinearity between variables.

		VIF	1/VIF
Residence	Rural	For reference	
	Urban	1.36	0.737626
Gender	Male	For reference	
	Female	2.49	0.401392
Health insurance	No health insurance	For reference	
	Basic health insurance	2.47	0.405459
	Other health insurance	2.51	0.397931
Education		1.39	0.72
Chronic disease		1.20	0.83
Type of chronic diseases	Hypertension	1.20	0.83
	Dyslipidemia	1.26	0.80
	Diabetes	1.13	0.88
	Malignancy	1.03	0.97
	Chronic lung disease	1.38	0.72
	Liver disease	1.07	0.94
	Heart attack	1.18	0.85
	Stroke	1.11	0.90
	Kidney disease	1.11	0.90
	Stomach or digestive disorders	1.16	0.86
	Emotional and mental problems	1.08	0.93
	Memory-related disorders	1.14	0.88
	Arthritis or rheumatism	1.19	0.84
	Asthma	1.34	0.74
Region	Eastern areas	For reference	
	Central areas	1.80	0.56
	Western areas	1.91	0.52
	Northeast areas	1.29	0.78
Family size		1.37	0.73
Marital status	Married	1.42	0.70
	Other	For reference	
Ethnic	Han	For reference	
	Other	1.06	0.94
Type of health service facility	General hospital	2.71	0.37
	Specialized hospital	1.76	0.57
	Chinese Medicine Hospital	For reference	
	Township Hospital	2.22	0.45
	Other	1.26	0.80
Received distance		1.04	0.96
Smoke	Now or have smoked	2.24	0.45
	Never smoke	For reference	
Alcoholic	Have drunk alcohol in the past year	1.16	0.86
	Haven’t drunk alcohol in the past year		
Pension	Yes	1.56	0.64
	No	For reference	
Mean VIF		1.49	

The VIF values range from 1.03 to 2.71, with an average VIF of 1.49, indicating that multicollinearity is generally not severe in the model. According to convention, VIF values below 10 are considered acceptable, suggesting that the model does not suffer from severe multicollinearity issues.

The reciprocal of VIF (1/VIF) provides a complementary perspective, where values closer to 1 indicate lower collinearity. Conversely, values closer to 0 suggest higher collinearity.

In summary, the VIF analysis indicates that while some variables show moderate collinearity, the overall model does not suffer from severe multicollinearity issues.

### 3.4 Benchmark regression results

#### 3.4.1 Analysis of influencing factors on older adult’s hospitalizational costs

The conducted linear regression analysis aims to explore the determinants of hospitalizational costs, shedding light on the intricate interplay between various demographic, socio-economic, and health-related factors.

As shown in Table 4, residence status emerged as a significant predictor, revealing that individuals residing in urban areas tend to incur higher inpatient out-of-pocket costs compared to their rural counterparts (*β* = 0.1550, *p* = 0.05). However, gender did not exhibit a statistically significant association with out-of-pocket costs, indicating no discernible difference between males and females (*β* = −0.1199, *p* = 0.25). Health insurance status displayed varied effects, with individuals possessing basic health insurance showing a marginal increase in costs (*β* = 0.2692, *p* = 0.30), while those with other health insurance experienced a non-significant decrease (*β* = −0.0808, *p* = 0.81).

**Table 4:** Older adults ‘hospitalizational costs linear regression model.

		Coef.	Std.Err.	*t*	*P* > |t|	[95% Conf. Interval]	
Residence	Rural	For reference					
	Urban	0.16	0.08	2.01	0.05	0.00	0.31
Gender	Male	For reference					
	Female	−0.12	0.10	−1.15	0.25	−0.32	0.08
Health insurance	No health insurance	For reference					
	Basic health insurance	0.27	0.26	1.04	0.30	−0.24	0.77
	Other health insurance	−0.08	0.33	−0.24	0.81	−0.74	0.57
Education		0.00	0.03	−0.11	0.92	−0.06	0.05
Chronic disease		0.20	0.18	1.14	0.25	−0.15	0.55
Type of chronic diseases	Hypertension	0.15	0.07	2.10	0.04	0.01	0.29
	Dyslipidemia	−0.06	0.08	−0.71	0.48	−0.21	0.10
	Diabetes	0.11	0.08	1.27	0.21	−0.06	0.27
	Malignancy	0.95	0.15	6.35	0.00	0.65	1.24
	Chronic lung disease	0.26	0.09	3.00	0.00	0.09	0.42
	Liver disease	0.26	0.11	2.34	0.02	0.04	0.47
	Heart attack	0.00	0.07	0.04	0.97	−0.14	0.15
	Stroke	0.15	0.09	1.67	0.10	−0.03	0.32
	Kidney disease	0.09	0.09	0.98	0.33	−0.09	0.27
	Stomach or digestive disorders	0.08	0.07	1.15	0.25	−0.06	0.22
	Emotional and mental problems	−0.11	0.16	−0.70	0.48	−0.43	0.20
	Memory-related disorders	−0.06	0.12	−0.56	0.58	−0.29	0.16
	Arthritis or rheumatism	−0.11	0.07	−1.58	0.11	−0.25	0.03
	Asthma	−0.13	0.11	−1.16	0.25	−0.34	0.09
Region	Eastern areas	For reference					
	Central areas	−0.41	0.10	−4.26	0.00	−0.59	−0.22
	Western areas	−0.38	0.09	−4.16	0.00	−0.56	−0.20
	Northeast areas	−0.21	0.15	−1.36	0.17	−0.51	0.09
Family size		0.13	0.13	0.07	1.92	0.06	0.00
Marital status	Married	0.08	0.09	0.87	0.38	−0.10	0.26
	Other	For reference					
Ethnic	Han	For reference					
	Other	0.02	0.13	0.16	0.87	−0.24	0.28
Type of health service facility	General hospital	0.20	0.11	1.80	0.07	−0.02	0.42
	Specialized hospital	0.38	0.15	2.45	0.01	0.08	0.68
	Chinese Medicine Hospital	For reference					
	Township Hospital	−1.02	0.14	−7.39	0.00	−1.29	−0.75
	Other	−0.66	0.22	−2.98	0.00	−1.10	−0.23
Received distance		0.00	0.00	0.00	5.01	0.00	0.00
Smoke	Now or have smoked	−0.17	0.10	−1.67	0.10	−0.36	0.03
	Never smoke	For reference					
Alcoholic	Have drunk alcohol in the past year	−0.10	0.05	−2.08	0.04	−0.19	−0.01
	Haven’t drunk alcohol in the past year	For reference					
Pension	Yes	For reference					
	No	0.06	0.10	0.67	0.51	−0.13	0.25
Constant		5.38	0.44	12.09	0.00	4.51	6.25

Among chronic diseases, malignancy (*β* = 0.9451, *p* < 0.01), chronic lung disease (*β* = 0.2558, *p* < 0.01), and liver disease (*β* = 0.2582, *p* = 0.02) were significantly associated with higher inpatient costs. Additionally, hypertension exhibited a significant positive association (*β* = 0.1523, *p* = 0.04). Regional disparities were evident, with individuals residing in central (*β* = −0.4074, *p* < 0.01) and western areas (*β* = −0.3814, *p* < 0.01) experiencing significantly lower inpatient costs compared to those in eastern areas. Type of health service facility also played a pivotal role, with individuals receiving care from township hospitals incurring significantly lower costs (*β* = −1.0191, *p* < 0.01) compared to those utilizing Chinese Medicine Hospitals. Similarly, those utilizing other health service facilities exhibited decreased expenses (*β* = −0.6607, *p* < 0.01). Notably, distance traveled to receive healthcare services demonstrated a significant positive association with inpatient expenses (*β* = 0.0009, *p* < 0.01), indicating that longer distances were associated with higher expenses.

Furthermore, individuals who had drunk alcohol in the past year exhibited significantly lower inpatient expenses (*β* = −0.1001, *p* = 0.04), while other demographic and socio-economic factors such as education level, marital status, and pension status did not display significant associations.

#### 3.4.2 Analysis of influencing factors on older adult’s hospitalizational burden

In this section, the ordinary least squares model was used to perform a regression analysis on the relationship between the self-payment burden (dependent variable) and the respective variables of the older adult population in China. Considering various demographic, socio-economic, and health-related factors. The results, summarized in the Table 5, provide insights into the associations between these factors and the financial burden borne by individuals in accessing healthcare services.

**Table 5:** Older adults ‘hospitalizational burden linear regression model.

		Coef.	Std.Err.	*t*	*P* > |t|	[95% Conf. Interval]	
Residence	Rural	For reference					
	Urban	−0.03	0.01	−2.38	0.02	−0.06	−0.01
Gender	Male	For reference					
	Female	0.00	0.02	−0.06	0.95	−0.04	0.03
Health insurance	No health insurance	For reference					
	Basic health insurance	−0.27	0.04	−6.07	0.00	−0.36	−0.18
	Other health insurance	−0.30	0.06	−5.23	0.00	−0.42	−0.19
Education		−0.01	0.01	−2.03	0.04	−0.02	0.00
Chronic disease		0.03	0.03	0.85	0.39	−0.03	0.09
Type of chronic diseases	Hypertension	0.00	0.01	−0.10	0.92	−0.03	0.02
	Dyslipidemia	0.01	0.01	1.10	0.27	−0.01	0.04
	Diabetes	−0.01	0.01	−0.89	0.37	−0.04	0.02
	Malignancy	0.04	0.03	1.44	0.15	−0.01	0.09
	Chronic lung disease	0.00	0.01	0.00	1.00	−0.03	0.03
	Liver disease	0.05	0.02	2.59	0.01	0.01	0.09
	Heart attack	−0.01	0.01	−0.97	0.33	−0.04	0.01
	Stroke	−0.02	0.02	−1.23	0.22	−0.05	0.01
	Kidney disease	−0.03	0.02	−1.66	0.10	−0.06	0.00
	Stomach or digestive disorders	0.01	0.01	0.70	0.49	−0.02	0.03
	Emotional and mental problems	−0.04	0.03	−1.31	0.19	−0.09	0.02
	Memory-related disorders	0.00	0.02	−0.24	0.81	−0.04	0.03
	Arthritis or rheumatism	−0.01	0.01	−0.72	0.47	−0.03	0.02
	Asthma	−0.02	0.02	−0.99	0.32	−0.06	0.02
Region	Eastern areas	For reference					
	Central areas	−0.05	0.02	−2.84	0.01	−0.08	−0.01
	Western areas	−0.04	0.02	−2.23	0.03	−0.07	0.00
	Northeast areas	0.00	0.03	−0.13	0.90	−0.06	0.05
Family size		0.03	0.01	2.25	0.01	0.05	0.05
Marital status	Married	−0.02	0.02	−1.30	0.19	−0.05	0.01
	Other	For reference					
Ethnic	Han	For reference					
	Other	0.04	0.02	1.69	0.09	−0.01	0.08
Type of health service facility	General hospital	0.04	0.02	2.00	0.05	0.00	0.08
	Specialized hospital	0.06	0.03	2.25	0.02	0.01	0.11
	Chinese Medicine Hospital	For reference					
	Township Hospital	−0.05	0.02	−2.00	0.05	−0.09	0.00
	Other	0.10	0.04	2.66	0.01	0.03	0.18
Received distance		0.00	0.00	0.00	3.57	0.00	0.00
Smoke	Now or have smoked	−0.03	0.02	−1.83	0.07	−0.07	0.00
	Never smoke	For reference					
Alcoholic	Have drunk alcohol in the past year	0.02	0.01	2.38	0.02	0.00	0.04
	Haven’t drunk alcohol in the past year	For reference					
Pension	Yes	For reference					
	No	0.10	0.02	5.76	0.00	0.06	0.13
Constant		0.28	0.07	3.96	0.00	0.14	0.41

The residence variable demonstrated a significant association with burden, with individuals residing in urban areas experiencing a slightly lower burden compared to their rural counterparts (*β* = −0.0317, *p* = 0.02). Regarding gender, no significant difference was observed in the burden between males and females (*β* = −0.0011, *p* = 0.95). Health insurance coverage emerged as a significant predictor of burden, with individuals having basic health insurance or other health insurance experiencing lower burden compared to those without health insurance (β = −0.2708, *p* < 0.01; *β* = −0.3028, *p* < 0.01, respectively), residents with other insurance have a lower burden of hospitalization than those with only basic insurance. Education level also showed a significant association, indicating that individuals with higher education tend to have lower burden (*β* = −0.0103, *p* = 0.04).

Among chronic diseases, liver disease was significantly associated with higher burden (*β* = 0.0486, *p* = 0.01), while patients with kidney disease had a lower burden (*β* = −0.0256 *p* = 0.10). Other chronic diseases did not show significant associations. Region-wise, individuals in central areas and the western areas exhibited a significantly lower burden compared to those in eastern areas (*β* = −0.0470, *p* = 0.01; *β* = −0.0354, *p* = 0.03). Moreover, family size was positively associated with burden (*β* = 0.0299, *p* = 0.01). Regarding marital status, being married was not significantly associated with burden compared to other marital statuses (*β* = −0.0206, *p* = 0.19). Ethnicity demonstrated a marginal association, with individuals of other ethnicities showing slightly higher burden compared to Han ethnicity (*β* = 0.0384, *p* = 0.09). Type of health service facility also played a role, with individuals receiving care from specialized hospitals or general hospitals experiencing higher burden compared to those utilizing Chinese Medicine Hospitals (*β* = 0.0602, *p* = 0.02; *β* = 0.0388, *p* = 0.05, respectively), conversely, those who were hospitalized in township hospitals had a lower burden. The distance traveled to receive healthcare services showed a significant association, with longer distances associated with slightly higher burden (*β* = 0.0001, *p* < 0.01).

Other factors such as smoking status, alcohol consumption, and pension status were also found to be associated with burden. Individuals who had not drunk alcohol in the past year and those receiving pensions experienced lower burden, while current smokers exhibited slightly higher burden.

### 3.5 Heterogeneity analysis

The heterogeneity analysis results were examined through a comparative analysis between the overall sample and its four subgroups representing distinct regions. This analysis aimed to assess the consistency of the relationships between independent variables and the natural logarithm of inpatient out-of-pocket costs across different geographical areas. In Table 4, the coefficients represent the estimated effects of independent variables on inpatient out-of-pocket costs, with significance denoted by asterisks (0.1*, 0.05 **, 0.01 ***). The Table 6 is organized into columns for the overall sample and each of the four subgroups (Eastern, Central, Northeast, and Western regions).

**Table 6:** Heterogeneity analysis of older adults ‘hospitalizational costs.

		All	Eastern	Central	Northeast	Western
Residence	Rural	For reference				
	Urban	0.16**	0.08	0.28**	−0.19	0.20*
Gender	Male	For reference				
	Female	−0.12	−0.17	−0.19	−0.07	−0.07
Health insurance No health insurance for reference	Basic health insurance	0.27	−1.02	0.61	3.16***	0.16
	Other health insurance	−0.08	−1.15	0.77	−0.87	−0.10
Education		0.00	−0.04	−0.06	0.13	0.00
Chronic disease		0.20	0.13	−0.03	−0.84	0.47
Type of chronic diseases	Hypertension	0.15**	0.01	0.00	0.47	0.20*
	Dyslipidemia	−0.06	−0.03	−0.16	0.19	−0.03
	Diabetes	0.11	−0.24	0.36**	0.56**	0.10
	Malignancy	0.95***	0.72**	1.63***	0.75	0.79***
	Chronic lung disease	0.26***	0.44**	0.34**	−0.04	0.20
	Liver disease	0.26**	0.11	0.36*	0.96**	0.13
	Heart attack	0.00	−0.03	0.12	−0.35	−0.02
	Stroke	0.15*	0.67***	0.04	−0.38	0.18
	Kidney disease	0.09	−0.10	0.13	−0.07	0.13
	Stomach or digestive disorders	0.08	−0.06	0.07	−0.41	0.18*
	Emotional and mental problems	−0.11	0.34	−0.20	1.16*	−0.29
	Memory-related disorders	−0.06	0.08	0.11	−1.02***	−0.02
	Arthritis or rheumatism	−0.11	0.01	−0.04	−0.23	−0.23*
	Asthma	−0.13	−0.31	−0.16	0.62	−0.12
Region	Eastern areas	For reference				
	Central areas	−0.41***	0.00	0.00	0.00	0.00
	Western areas	−0.38***	0.00	0.00	0.00	0.00
	Northeast areas	−0.21	0.00	0.00	0.00	0.00
Family size		0.13*	0.22	0.22	−0.10	0.08
Marital status	Married	0.08	−0.21	0.00	0.46	0.20
	Other	For reference				
Ethnic	Han	For reference				
	Other	0.02	−0.19	0.75	−0.61	0.00
Type of health service facility	General hospital	0.20*	0.00	0.02	0.07	0.36**
	Specialized hospital	0.38**	0.40	0.14	0.02	0.40*
	Chinese Medicine Hospital	For reference				
	Township Hospital	−1.02***	−0.95***	−1.18***	−1.60**	−0.95***
	Other	−0.66***	−0.65	−1.40***	0.07	−0.44
Received distance		0.00***	0.00***	0.00**	0.00*	0.00***
Smoke	Now or have smoked	−0.17*	−0.40*	−0.07	0.22	−0.17
	Never smoke	For reference				
Alcoholic	Have drunk alcohol in the past year	−0.10**	0.03	−0.10	−0.25	−0.15**
	Haven’t drunk alcohol in the past year	For reference				
Pension	Yes	For reference				
	No	0.06	−0.15	−0.03	0.27	0.26*
Constant		5.38***	7.48***	5.14***	3.47**	4.29***

Urban residence exhibits a positive coefficient in the overall sample and Central region. The female gender generally shows negative coefficients across all subgroups and the overall sample. Basic health insurance demonstrates a positive coefficient in the overall sample and several subgroups, with no significant results in the Northeast region. Other health insurance also shows varied effects across subgroups. Generally, education exhibits negative coefficients across all subgroups and the overall sample, although not always statistically significant.

The presence of chronic diseases generally shows positive coefficients across subgroups and the overall sample. The coefficient for hypertension patients suggests a slight increase in inpatient expenses. The coefficient for diabetes patients suggests a potential increase in inpatient expenses, notably significant in the Central and Western regions within the overall sample. The coefficient for malignancy indicates a significant positive effect on inpatient expenses, particularly in the Eastern region. The coefficient for chronic lung disease patients suggests a potential increase in inpatient costs, especially significant in the Eastern region within the overall sample. The coefficient for liver disease patients indicates a positive impact on inpatient expenses, especially significant in the Central and Western regions. The coefficient for stroke patients suggests a potential increase in inpatient expenses, particularly significant in the Eastern region.

The coefficient for family size is positive in the overall sample and most subgroups. Coefficients vary across different types of health service facilities, with significant effects observed for specialized hospitals in the overall sample and Township Hospitals in various subgroups. Generally, received distance exhibits positive coefficients, with statistically significant results in all subgroups. People who have smoked cigarettes and those who have drunk alcohol in the past year usually show a negative correlation. People with pensions generally have higher costs, although the level of significance varies among different subgroups.

Overall, the coefficients demonstrate consistency in directionality across subgroups and the overall sample for many independent variables. However, there are also notable variations in significance levels and magnitudes of effects.

The Table 7 presents the results of robustness tests for the burden across different subgroups and the overall sample. The coefficients represent the estimated effects of various independent variables on the dependent variable, with emphasis on the consistency and significance of coefficients across different subgroups and the entire sample.

**Table 7:** Heterogeneity analysis of older adults ‘hospitalizational burden.

		All	Eastern	Central	Northeast	Western
Residence	Rural	For reference				
	Urban	−0.03**	−0.03	−0.03	−0.12*	−0.02
Gender	Male	For reference				
	Female	−0.00	0.02	−0.03	−0.07	0.02
Health insurance	No health insurance	For reference				
	Basic health insurance	−0.27***	−0.49***	−0.23**	0.25	−0.32***
	Other health insurance	−0.30***	−0.50***	−0.17	0.17	−0.42***
Education		−0.01**	0.00	−0.02**	0.01	−0.01
Chronic disease		0.01	0.06	0.02	−0.12	−0.01
Type of chronic diseases	Hypertension	0.00	0.01	0.01	0.01	−0.01
	Dyslipidemia	0.01	0.00	−0.01	0.02	0.03
	Diabetes	−0.01	−0.03	0.02	−0.02	−0.02
	Malignancy	0.04	0.05	0.06	−0.10	0.04
	Chronic lung disease	0.00	−0.01	0.03	0.03	−0.02
	Liver disease	0.05***	0.00	0.04	0.04	0.09***
	Heart attack	0.00	−0.01	0.00	−0.08	−0.01
	Stroke	−0.02	0.00	−0.04	−0.06	−0.01
	Kidney disease	−0.03*	−0.05	−0.01	−0.04	−0.02
	Stomach or digestive disorders	0.01	−0.01	0.00	−0.03	0.01
	Emotional and mental problems	0.00	0.02	0.00	−0.05	−0.01
	Arthritis or rheumatism	0.00	−0.02	0.00	0.00	0.00
	Asthma	−0.02	−0.02	−0.06*	0.07	0.00
Region	Eastern areas	For reference				
	Central areas	−0.05***	0	0	0	0
	Western areas	−0.03**	0	0	0	0
	Northeast areas	−0.00	0	0	0	0
Family size		0.03**	0.05*	0.06**	0.10*	0.01
Marital status	Married	−0.02	−0.04	−0.08**	−0.06	0.03
	Other	For reference				
Ethnic	Han	For reference				
	Other	0.04*	0.02	−0.01	−0.10	0.07**
Type of health service facility	General hospital	0.04**	−0.04	0.06	−0.02	0.05*
	Specialized hospital	0.06**	0.07	0.10*	−0.06	0.02
	Chinese Medicine Hospital	For reference				
	Township Hospital	−0.05**	−0.10*	−0.04	−0.14	−0.03
	Other	0.10***	0.03	0.12	−0.11	0.12**
Received distance		0.00***	0.00**	0.00**	0.00*	0.00
Smoke	Now or have smoked	−0.03*	−0.03	−0.03	−0.05	−0.03
	Never smoke	For reference				
Alcoholic	Have drunk alcohol in the past year	0.02**	0.01	0.01	0.03	0.03**
	Haven’t drunk alcohol in the past year	For reference				
Pension	Yes	For reference				
	No	0.10***	0.15***	0.05	0.01	0.13***
Constant		0.58***	0.67***	0.58***	0.49	0.52***

In urban areas, there is a statistically significant negative effect on the burden, which is consistent across all regions. However, the effect is only significant in the Northeast at a lower level of significance. Females generally have a negative effect on the burden, but the effect is not statistically significant across all regions and the entire sample. Both basic health insurance and other health insurance have significant negative effects on the burden, consistent across all regions and the entire sample except for the northeast. Higher levels of education have a significant negative effect on the burden, consistent across all regions and the entire sample except for the northeast.

Liver disease is usually positively correlated with hospitalization burden, while kidney disease has a negative coefficient in both the total sample and subgroups and has some significance. A higher family size generally leads to a higher burden, with significant positive effects in some regions and the entire sample. Belonging to a non-Han ethnicity has varying effects on the burden across regions and the entire sample.

The type of health facility has varying effects on the burden across regions and the entire sample. Compared to Chinese Medicine Hospitals, both Specialized Hospitals and other types of health service facilities exhibit positive coefficients, except in the Northeast region. Township Hospitals, on the other hand, consistently show negative coefficients across all regions and the overall sample. There is a statistically significant positive effect of distance on the burden, consistent across all regions and the entire sample.

Individuals who have smoked tend to have lower burden, whereas those who have consumed alcohol in the past year exhibit higher burden. Moreover, individuals without pension tend to incur higher costs for hospitalization. Furthermore, some coefficients exhibit varying levels of significance across regions, highlighting the nuanced impact of these factors on healthcare expenses.

Overall, the results highlight the importance of considering various factors and their nuanced effects on the burden, with some consistent patterns across regions and the entire sample.

## 4 Discussion

### 4.1 Social factors

#### 4.1.1 Health insurance

Insurance has no significant impact on hospitalization costs, but for older adult individuals without insurance, those with basic medical insurance and additional insurance coverage beyond basic medical insurance experience significantly reduced burden, with those possessing additional insurance experiencing even greater reductions.

Firstly, basic medical insurance typically provides some degree of coverage for medical expenses, which can reduce residents’ economic burden in seeking medical care, increase accessibility to healthcare services, and meeting residents’ medical service needs. It is particularly crucial for older adult individuals who often face more health issues and higher medical expenses (28). However, basic medical insurance may not cover all types of medical services and expenses, especially for expensive treatments and medications. In such cases, having additional insurance (such as commercial medical insurance or supplementary medical insurance) may further reduce older adult individuals’ medical expense burden (29).

#### 4.1.2 Pension

Older adult individuals with pensions tend to incur greater burden. Research has indicated that receiving a pension can increase hospitalization costs by 2,538 yuan, and other medical expenses by 1,203 yuan (30). Individuals with pensions may be more inclined to seek expensive medical services and treatments because they have financial support to cover these expenses. In contrast, those without pensions or with less economic support may avoid or delay medical treatment due to costs, leading to lower medical expenses.

### 4.2 Medical factors

#### 4.2.1 Type of health service facility

Compared to traditional Chinese medicine hospitals, comprehensive and specialized hospitals tend to incur higher expenses and burden, while township hospitals typically have lower costs.

Comprehensive hospitals typically offer a wide range of medical services and comprehensive scope, these hospitals often possess more medical resources and specialized personnel capable of handling various complex medical conditions. This comprehensive approach may result in higher medical expenses for patients seeking treatment at comprehensive hospitals.

Specialized hospitals, on the other hand, focus on specific types of medical services such as cardiac care and cancer treatment. Physicians and equipment at specialized hospitals are usually tailored to address specific diseases. In some cases, specialized hospitals may offer more cost-effective treatment options. However, for conditions outside their specialty, patients may need to be referred to other hospitals, potentially increasing overall medical costs and burden.

Township hospitals may invest less in medical equipment and human resources, leading to lower unit service costs. Additionally, due to their smaller scale, township hospitals may find it easier to control costs. These hospitals mainly provide basic medical services and may have limited capabilities for treating complex diseases, thus resulting in lower hospitalization costs.

#### 4.2.2 Received distance

The geographical location of medical institutions directly affects patients’ medical expenses and burden. In real-life scenarios, not all patients opt for the nearest healthcare facility. This is because not every medical institution is equipped to handle a wide range of illnesses. Therefore, the distance or time required to reach the closest medical service point must consider the varying capabilities of different healthcare providers (31–33). For patients diagnosed with complex and critical conditions, they may choose to travel to more distant hospitals in pursuit of superior medical care, even though the cost of treating these symptoms is inherently not low.

### 4.3 Family factor

Older adult individuals with a larger number of family members tend to incur higher hospitalization costs and burden. Firstly, a larger family size may potentially equate to a higher household income, which could enable them to allocate greater financial resources towards hospitalization costs (34). Secondly, larger family sizes may face challenges in coordinating care and making decisions, potentially leading to fragmented or inefficient healthcare utilization, which contribute to increased hospitalization costs (35).

### 4.4 Personal factors

#### 4.4.1 Education

The impact of education level on hospitalization costs is not conspicuous; however, higher-educated older adult individuals tend to bear lower hospitalization burden. For the older adult population, higher education levels may be linked to better understanding and utilization of health insurance policies and benefits. This can result in more effective utilization of the healthcare system and proper utilization of healthcare resources, ultimately reducing hospitalization rates and burden among older adult individuals with higher education levels (36, 37).

#### 4.4.2 Chronic diseases

Firstly, chronic diseases such as hypertension, malignancy, chronic lung disease, liver disease, and stroke significantly increase hospitalization costs. These conditions typically require long-term, sustained treatment and care, often involving costly medications and medical procedures. For instance, cancer treatment may entail chemotherapy, radiation therapy, targeted therapy, among others (38). Similarly, patients with chronic lung disease and stroke may require interventions such as ventilator support, rehabilitation (39).

However, the impact of liver disease and kidney disease on hospitalization burden may vary. Some studies suggest that liver disease may lead to increased medical costs because patients may utilize more emergency care hospitalization (40). On the other hand, the effect of kidney disease on medical costs may differ in certain circumstances. This could be because after receiving treatments such as dialysis, the condition of patients with kidney disease may be partially controlled, thereby reducing the occurrence of other complications and related medical burden (41).

#### 4.4.3 Personal habits

Smokers tend to have lower hospitalization costs and burden, while individuals who consumed alcohol in the past year tend to have higher burden and lower costs. In general, regular smokers have a longer hospital stay than those who never smoke regularly (42), there will be greater hospitalization costs and burden. However, smokers may reduce their demand for and utilization of medical services due to poorer health conditions. This reduction may be due to barriers or biases in accessing health services among smokers, or because they may lack sufficient financial resources to cover medical expenses or may lack adequate medical insurance coverage. Another study found that smokers are typically associated with lower socioeconomic status (43).

There is a negative correlation or U-shaped relationship between alcohol consumption levels and health service use (44), abstainers, in particular, are more likely to use health care services than low-risk alcohol consumers (45), A 4-year prospective study by Anzai et al. (46) found, Lifelong abstinent alcoholics and alcoholics have higher inpatient health care utilization. A higher proportion of people were likely to be light and moderate drinkers in the sample, as a result, fewer people will take advantage of inpatient services and incur lower costs.

#### 4.4.4 Ethnic

The study by Qiu Yulin and Zhang Zhongchao indicates that the probability of poverty due to illness in ethnic minority areas of Guizhou is significantly higher than in non-ethnic areas. Notably, within non-ethnic regions, the poverty incidence in areas with ethnic minority autonomy is even higher. This suggests that ethnic differences may be associated with economic resources and medical insurance coverage, thereby affecting the burden of hospitalization costs. If older adult individuals from other ethnic groups have lower medical insurance coverage rates or smaller insurance reimbursement ratios, they may have to bear a greater burden of inpatient medical expenses (47).

### 4.5 Geographic factors

#### 4.5.1 Residency

Research indicates that hospitalization costs for urban residents are generally higher than those for rural residents, possibly due to higher medical service costs and more advanced medical technologies in urban areas. Urban older adult individuals are more likely to receive regular health check-ups (48), which facilitate early detection of chronic diseases. The prevalence of chronic diseases is higher among urban older adult individuals compared to rural counterparts (49, 50). However, the hospitalization burden for urban residents is relatively lower, possibly attributed to the greater benefits in health insurance coverage in urban areas (51).

#### 4.5.2 Region

Compared to the eastern region, the central and western regions of China tend to have lower hospitalization costs and burden. Firstly, the eastern region of China is characterized by its abundant medical resources, which include a higher concentration of top-tier hospitals, state-of-the-art medical equipment, and a more extensive network of medical professionals. This abundance may lead to more costly medical treatments in the area, consequently driving up hospitalization costs. In contrast, the central and western regions face a scarcity of medical resources, which could result in more affordable medical expenses (52, 53). Secondly, income plays a significant role in the hospitalizational costs and the financial burden of hospitalizations for the older adult (54). The eastern region, with its higher economic development, provides residents with higher income levels. This increased financial capacity may enable residents to bear higher medical expenses, further contributing to the elevated hospitalization costs in the region. On the other hand, the central and western regions, where economic development is relatively lower, may see residents opting for more cost-effective medical services due to their limited ability to pay. This could lead to a reduction in hospitalization costs in these areas.

## 5 Conclusion

This study utilizes data from the China Health and Retirement Longitudinal Study (CHARLS) spanning from 2011 to 2018 to investigate the costs and burden of hospitalization for the older adult in China from a micro-perspective. It also explores the factors that may influence these costs and burden. Empirical findings reveal that the average out-of-pocket expenditure for hospitalization among Chinese older adult in 2018 was $1,199.24, with a burden ratio of 0.5. Several factors were identified to significantly increase the out-of-pocket hospitalization costs, including urban, hypertension, malignancy, chronic lung disease, liver disease, and stroke as prevalent chronic conditions, family size, general hospital, specialized hospital, received distance, and absence of pension. Conversely, certain factors were found to significantly reduce the hospitalization costs for the older adult, including residence in central and western regions; utilization of township and other hospitals; and a history of smoking or drinking. In terms of the burden of hospitalization for the older adult, conditions such as liver disease, family size, ethnic minorities, health service facility other than township hospitals, received distance, a history of alcohol consumption within the past year, and lack of a pension were significant contributors to increased burden. On the other hand, factors such as urban, basic health insurance and other health insurance, level of education, kidney disease, central and western regions, township hospitals, and a history of smoking were found to significantly alleviate the burden of hospitalization for the older adult. This research provides valuable insights into the financial implications of hospitalization for China’s aging population and highlights the need for targeted policies to mitigate the economic burden on this vulnerable group.

In view of the above situation, we recommend the following targeted strategies: Continue to expand medical insurance coverage to ensure more equitable access to healthcare services. Actively promote commercial insurance options that can complement basic health insurance, providing additional financial protection against high medical costs. Strengthen primary medical services to reduce the burden on higher-level hospitals, thereby potentially lowering the overall cost of hospitalization. Implement policies that promote equity in health resources and work to reduce regional disparities in healthcare availability and quality. Introduce targeted interventions for vulnerable groups, such as medical cost subsidies, special insurance schemes, and financial assistance programs, to alleviate the financial stress associated with hospitalization. Acknowledge and address ethnic differences in healthcare by providing language translation services and culturally tailored health education materials to ensure inclusive and effective healthcare communication.

This study acknowledges certain limitations in its design and scope. Firstly, the analysis was based solely on data from the 2018 China Health and Retirement Longitudinal Study (CHARLS), which restricts the ability to conduct a comparative analysis of the hospitalization hospitalizational costs and burden for the older adult across different years, thereby precluding the observation of trends and changes over time. Secondly, the hospitalization cost data collected in the 2018 CHARLS survey encompassed only the expenses paid to the hospital itself, excluding additional costs such as the wages of accompanying caregivers, transportation fees for the patient or family members, and accommodation expenses. However, it did include the cost of hospital room charges. As a result, this study was unable to analyze non-medical expenses associated with hospitalization. Furthermore, the cost data only distinguished between hospitalizational costs and did not break down into specific categories such as material fees, medication costs, and room charges, which limits the detailed analysis of the composition of hospitalization costs. Lastly, this research is retrospective in nature and does not incorporate any predictive modeling or forecasting. The findings are based on the exam ethnic of past data without projection into future trends or outcomes.

These limitations should be taken into account when interpreting the results of this study and when considering its implications for policy and practice. Future research efforts may address these gaps by incorporating multi-year data, a more comprehensive range of hospitalization costs, and potentially predictive analytics to provide a more robust understanding of the financial burden faced by the older adult during hospitalization.

## Data availability statement

The original contributions presented in the study are included in the article/Supplementary material, further inquiries can be directed to the corresponding author.

## Ethics statement

The studies involving humans were approved by Biomedical Ethics Review Committee of Peking University. The studies were conducted in accordance with the local legislation and institutional requirements. The participants provided their written informed consent to participate in this study.

## Author contributions

SH: Writing – original draft, Writing – review & editing. YB: Writing – review & editing.
